# External hemorrhoidal disease in child and teenage: Clinical presentations and risk factors

**DOI:** 10.12669/pjms.35.3.442

**Published:** 2019

**Authors:** Turan Yildiz, Dilek Bingol Aydin, Zekeriya Ilce, Aysel Yucak, Erol Karaaslan

**Affiliations:** 1 *Turan Yildiz Department of Pediatric Surgery, Inonu University, Turgut Ozal Medical Center, Malatya, Turkey*; 2 *Dilek Bingol Aydin Department of Pediatric, Sakarya University Education and Research Hospital, Sakarya, Turkey*; 3 *Zekeriya Ilce Department of Pediatric Surgery, Sakarya University School Medicine, Sakarya, Turkey*; 4 *Aysel Yucak Department of Pediatric Surgery, Sakarya University School Medicine, Sakarya, Turkey*; 5 *Erol Karaaslan Department of Anestesiology and Reanimation, Inonu University, Turgut Ozal Medical Center, Malatya, Turkey*

**Keywords:** Child, Diagnosis, External hemorrhoid, Risk factors, Treatment

## Abstract

**Objective:**

Hemorrhoidal disease (HD), though mostly seen in adults, has recently emerged as a common problem among children. However, the diagnosis and treatment of HD in children is mostly based on the data obtained in adult studies. In this study, we aimed to evaluate risk factors, diagnostic and treatment modalities in the children diagnosed with external HD.

**Methods:**

The study was conducted at Sakarya University Medical School Pediatric Surgery Department between January 2012 and July 2018. We reviewed children who were diagnosed as having HD at Pediatric Surgery clinic. Age, gender, presenting symptoms, physical examination findings, risk factors, and treatment outcomes were evaluated for each patient.

**Results:**

The study included 56 patients with a mean age of 140.8±45.2 months. The patients comprised 48 (85.7%) boys and 8 (14.3%) girls. Constipation and a positive family history were the most common risk factor (n=33; 58.9%, n=29; 51.8%, respectively). Conservative treatment was performed in 53 (94.6%) patients. Recurrence was observed in 5 (8.9%) and skin tag was detected in 6 (10.7%) patients.

**Conclusions:**

External HD mostly occurs in boys in their second decade of life. Positive family history and constipation were the most common risk factors in our patients. Conservative treatment is sufficient for the management of external HD in children because of its low recurrence rates,

## INTRODUCTION

Hemorrhoidal disease (HD), also known as symptomatic hemorrhoids, is more common in adults while its incidence in children has been reported to increase.^1^,^2^

Hemorrhoids are vascular submucosal cushions located in the anal canal. These cushions are also known as corpus cavernosum recti and function as erectile cushions that aid in defecation and prevent stool leakage. The cushions become dilated and thrombosed as a result of the loosening of the connective tissues and protrude out of the anal canal, thereby becoming symptomatic and resulting in HD.^3^,^4^ There are three main bundles of hemorrhoidal tissue that are located at the left lateral, right anterolateral, and right posterolateral positions. Symptomatic hemorrhoids are named internal or external depending on their location. External hemorrhoids are localized distal to the dentate line and covered by anoderm, whereas internal hemorrhoids are localized proximal to the dentate line and covered by rectal or anorectal mucosa.^2^,^3^,^5^

Although there is a large body of literature on HD in adults, there are a limited number of studies reporting on HD in children and most of these studies include either case reports or studies with small patient series.^2^,^6^,^7^ Moreover there is also limited literature about diagnosis and treatment experience on pediatric external HD patients. Therefore, the data obtained via adult studies are often used for the child patients with HD that present to departments including pediatric diseases, pediatric gastroenterology, and pediatric surgery. In this study, we aimed to evaluate the epidemiological features, risk factors, and diagnostic and treatment characteristics in the children diagnosed with external HD.

## METHODS

The study included a patient group of 56 children who were diagnosed with external HD in Sakarya University Medical School Pediatric Surgery Department between January 2012 and July 2018 and a control group of 40 children who presented to the Pediatric Diseases Department due to respiratory tract infections and had no sign of HD. A written informed consent was obtained from each participant/guardian. Patients with incomplete medical records were reached by telephone and their records were completed, for which an approval was obtained from local ethics committee of Sakarya University (Approval no: 71522473/050.01.04/272). The study was conducted in accordance with the principles of the Declaration of Helsinki. However, patients with incomplete medical records and those who were detected with internal HD and were lost to follow up were excluded from the study. Age, gender, risk factors, presenting symptoms, rectal examination findings, type of HD, radiological and laboratory tests, treatment methods, and follow-up data were evaluated for each patient.

### Risk factors

The risk factors identified for adult patients in the literature were evaluated in each participant.^8^ These factors included constipation, type of toilet, obesity, and family history.

The diagnosis of constipation was made according to Rome III criteria.^9^ Medical records were also reviewed for hematologic, biochemical, and abdominal radiographs were reviewed for the presence of constipation. The types of toilet used by the patients were questioned with telephone. The toilet types classified into two groups; bidet and squat. The presence of HD was investigated in the first degree relatives of the patients to identify hereditary transmission. Accordingly, the patients were categorized into two groups, the patients with positive family history and the patients with negative family history.

Obesity was evaluated by calculating the body mass index (BMI) for each patient based on the body height and weight of the patients obtained from the medical records. For patients between 5 and 19 y of age, BMI 14.5–24.9 k/m^2^ was recognized as normal, BMI 25-29.9 kg/m^2^ was recognized as overweight, and BMI ≥30 kg/m^2^ was recognized as obese.^10^

Hemorrhoid examination was performed in children who had anorectal region complaints (swelling, rectal pain or difficulty sitting, mucus discharge, itching, bleeding). For this purpose, we performed physical examination, rectal touche examination and rectoscopy. The swelling that localized on distal of dentate line and covered by anoderm was diagnosed as external hemorrhoid. The painful and bluish-colored lump at the anal region was defined as trombosed external hemorrhoid.

Each patient underwent conservative or surgical treatment depending on presenting symptoms. Medical treatment was applied to all patients except the thrombosed hemorrhoids who were hospitalized in the first 72 hours. An anticonstipation diet, consuming plenty of water, using laxatives in the presence of constipation, taking local painkillers, and to perform a hot sitz bath were advised in patients younger than 12 years old. Venotonic drugs were added to the treatment in patients older than 12 years old. 14-day medical treatment was applied to the patients. In control relief of hemorrhoidal symptoms was considered as cure.

### Surgical treatment

The surgical treatment was performed in patients who were presented in the hospital with thrombosed hemorrhoids within first 72 hours. Thrombectomy was performed under general anesthesia. Postoperative complications were recorded for each patient. Patients were followed up at postoperative day 10 and months one and three.

### Statistical Analysis

The analyses were performed using a commercial software package (IBM SPSS Statistics, Version 22.0., IBM Corp, Armonk, NY, USA). Two independent samples *t*-tests were used to compare continuous data between the groups. Continuous variables were expressed as mean ± standard deviation (SD). Categorical variables were compared using the Chi-square test. Correlations between the variables were analyzed using Pearson’s Correlation Coefficient.

## RESULTS

The characteristics and the statistical data of the patients are shown in Table-I. The 56 patients included 48 (85.7%) boys and 8 (14.3%) girls with a mean age of 140.8±45.6 months and the control group included 22(55%) boys and 18(45%) girls with a mean age of 130.07±38.07 months. Of the 56 patients, 38 (67.9%) were aged older than 10 years. Although no significant difference was found with regards to age (*p*=0.229), a significant difference was found in terms of gender (*p*=0.001) between the two groups.

**Table-I:** Comparison of patients’ characteristics between hemorrhoids and control groups.

Characteristics		Hemorrhoids (n=56)	Controls (n=40)	P
Age (months)		140.8±45.6	130.07±38.07	0.229
Gender	Female	8 (14.3)	18 (45)	0.001
	Male	48 (85.7)	22 (55)	
Toilet	Squat	27 (48.2)	28 (70)	0.033
	Bidet	29 (51.8)	12 (30)	
Family History	Positive	29 (51.8)	7 (17.5)	0.001
	Negative	27 (48.2)	33 (82.5)	
Constipation	Positive	33 (58.9)	10 (25)	0.001
	Negative	23 (41.1)	30 (75)	
BMI	Normal	53 (94.6)	38 (95)	0.938
	Obese	3 (5.4)	2 (5)	

Data are shown as mean ± standard deviation and n (%)

### Risk factors

Constipation was detected in 33 (58.9%) of the patients, all of whom underwent treatment. A significant difference was found between the two groups in terms of constipation (*p*=0.001).A positive family history was found in 29 (51.8%) of the patients and a significant difference was found between the two groups (*p*=0.001). However, no correlation was found between the patients with a positive family history and the patients with constipation (*p*=0.118).

Of the 56 patients, 29 (51.8%) were using a bidet toilet and 27 (48.2%) were using a squat toilet. The patients were using the bidet toilet more frequently compared to the control subjects (*p*=0.033). No significant correlation was found between constipation and the type of toilet (*p*=0.119).

On the other hand, 3 (5.3%) patients were obese and 2 (3.5%) were overweight, although no significant difference was found between the two groups (*p*=0.938). ***Symptoms:*** The presenting symptoms of the patients are shown in Table-II. The most common presenting symptom was a breast-like swelling in the anorectal region (n=30; 53.5%), followed by rectal pain or difficulty sitting (n=16; 28.5%), nonbleeding mucus discharge (n=14; 25%), itching (n=8; 14.2%), bleeding (n=5; 8.9%), and any combination of these symptoms (n=17; 30.1%).

**Table-II:** Presenting symptoms.

	Patients (n=56)	

	n	Percent (%)
Lump near anus	30	53.5
Rectal pain or difficulty sitting	16	28.5
Non bleeding mucus discharge	14	25
Itching	8	14.2
Bleeding	5	8.9

### Physical Examination

**Fig.1:**
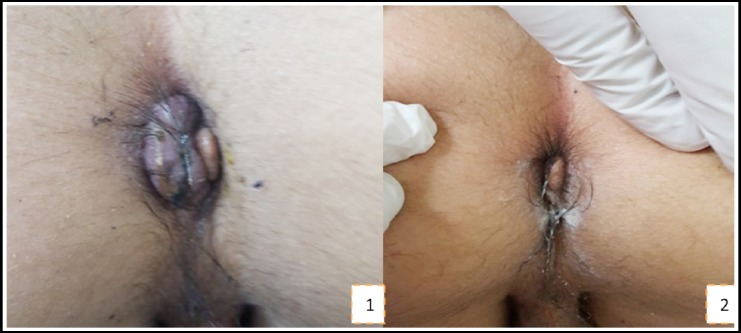
Appearance of external hemorrhoids and control examination. 1. Thrombosed hemorrhoid in 12 year old child, 2. First month follow up appearance of patient.

Physical examination was normal except for the rectal area where prolapsed hemorrhoids were seen (Fig.1). Thrombosed hemorrhoids were detected in 13 (23.2%) patients. During rectal touche examination, rectoscopy was performed in two patients on the suspicion of rectal polyp and internal hemorrhoid was detected in one patient. Thrombectomy(surgical treatment) was performed in 3(5.3%) patients. Medical treatment was performed in 53(94.7%) patients. In the 27 (48.2%) patients older than 12 years, venotonic drugs were added to the medical treatment. At month dthree, the external hemorrhoids became symptomatic again in 5 (8.9%) patients and skin tag was detected in 6 (10.7%) patients.

## DISCUSSION

Hemorrhoidal disease (HD) is common in adults.^11^ In contrast, it is rarely seen in childhood patients although the incidence of HD in children and adolescents has been shown to increase.^1^,^2^,^12^ Literature indicates that there is no gender preponderance for external hemorrhoids in adult population^13^,^14^; however, most of the children reported with HD are boys.^2^,^6^,^7^,^9^ Similarly, in our study, we also noted that external HD was more common in boys, which could be attributed to the finding reported by adult studies that the anal canal in males is longer than females.^4^ Additionally, we consider that this difference could play a role in the manifestation of symptomatic hemorrhoids.

Grossmann et al.^2^ reported a mean age of 4.5 years in the children with internal hemorrhoids. External hemorrhoids, however, have been mostly reported in young adults.^15^ Hemorrhoids are normally held in the anal canal by Treitz’s muscle and longitudinal ligaments. Relaxation of these structures with aging is considered to increase the incidence of HD.^3^ This occurrence, according to Jamshidi et al.^16^, explains the reason as to why HD is not commonly seen in children aged below 10 years. However, in our study, 32.1% of the patients were aged below 10 years and there are also several case reports in the literature presenting children aged below 10 years.^2^,^6^,^7^

A number of risk factors have been described for HD in adults. These factors mostly include age, pregnancy, abdominal obesity, depression, constipation, diarrhea, sitting for long periods of time on the toilet, lack of fiber in diet, inadequate hydration, alcohol abuse, spicy foods, and conditions leading to increased intraabdominal pressure.^11^ Constipation is the most common risk factor among children with HD.^11^,^12^ Constipation, together with sitting for long periods of time on the toilet, leads to increased intraabdominal pressure, thereby leading to HD.^1^,^3^ In our study, constipation was present in 58.9% of the patients. Moreover, although our study had a retrospective design and no evaluation was performed for the durations of toilet sitting in the study, a correlation was found between prolonged sitting and toilet type. Meaningfully, the incidence of HD was higher in the patients using a bidet (European) toilet compared to those using a squat (Asian) toilet, which could be attributed to the longer toilet sitting periods in the bidet toilet. On the other hand, a high rate of positive family history (*i.e*. presence of HD in at least one first-degree relative) was found in our patients (51.8%). Considering that this positivity could be associated with similar dietary habits and constipation among the family members, we performed a correlation analysis, which showed no correlation. In addition to family history, congenital weakness of the vein wall has also been implicated in the pathophysiology of HD.^17^ In our study, the high number of patients with a positive family history implies that HD could be associated with congenital weakness of the vein wall. Nevertheless, since there is no study reporting on the effect of family history in HD, further large-scale studies are needed to substantiate our finding.

External HD often manifests with a pain associated with the thrombosis of hemorrhoids and a palpable mass in the anal area.^1^,^2^,^4^,^11^,^13^,^17^ In our patients, an anal mass was the most common presenting symptom, which was accompanied by itching, mucus discharge, and bleeding. Although bleeding is a common symptom in internal HD, it results from the ulceration of the thrombosed hemorrhoids in external HD.^4^

External hemorrhoids are often revealed by external inspection.^3^ Moreover, while external hemorrhoids can be exposed by pulling aside the gluteal muscles, internal hemorrhoids can be seen via anoscopy. Rectal touche examination provides information on the tone of the anal sphincter, masses, and tenderness. In the cases with anal tenderness, this examination can be performed under anesthesia by inserting a Foley catheter in the anus and withdrawing it after inflating the catheter balloon.^1^,^3^,^7^ In our study, the patients were diagnosed by physical examination and rectoscopy was performed in 2 patients on the suspicion of rectal polyp during rectal touché examination.

Conservative treatment has been shown to provide effective outcomes in 50% of the patients with HD.^18^ Greenspon et al.^13^ performed conservative treatment in 51.5% of the patients with thrombosed external hemorrhoids and Oh^15^ reported that all the patients recovered with conservative treatment. Common conservative treatment options include modulation of fluid intake, diet, stool softeners, sitz baths, local hygiene, prevention of constipation and diarrhea, and oral and topical agents.^3^,^19^ In thrombosed external hemorrhoids, the conservative treatment is continued if it leads to thrombus resorption and pain relief.^13^ In adult patients, topical agents including steroids, anesthetic agents, and antiseptic agents have been used for the resolution of symptoms.^18^ Moreover, topical calcium channel blockers have also been used in the treatment of thrombosed external and internal hemorrhoids. Additionally, oral treatment often includes venotonic drugs^3^, which can increase vascular tone, reduce venous capacity, decrease capillary permeability, facilitate lymphatic drainage, also have anti-inflammatory effects, all of which lead to improvement in bleeding, pruritus, and general symptoms.^18^ To our knowledge, there are a limited number of studies investigating the treatment of childhood HD and these limited data indicate that conservative treatment is the method of choice in children with HD, as in adult patients.^1^

### Limitations of the study

First, the study had a retrospective design and the statistical power of the study was weak for performing a detailed risk factor analysis. Moreover, the study had no data regarding internal hemorrhoids. Therefore, further prospective studies involving data on internal hemorrhoids in children are needed to substantiate our findings.

HD is gradually becoming more common in children and most of the children with HD present with external HD which mostly occurs in boys in their second decade of life. A positive family history and constipation were the most common risk factors in our patients and palpable anal mass and pain were the most common presenting symptoms. We conclude that, conservative treatment, given its low recurrence rates, should be sufficient for the management of external HD.

### Authors Contribution

**TY:** conceived, designed and did statistical analysis, editing of manuscript.

**DBA, AY:** did data collection and manuscript writing.

**ZI:** did designed, review and final approval of manuscript.

**EK:** did editing of manuscript.
